# Trends in alcohol use disorder symptoms among U.S. adults disaggregated by sex, race, and age

**DOI:** 10.1007/s00127-025-02910-7

**Published:** 2025-04-30

**Authors:** Jessica K. Perrotte, Priscilla Martinez, Yessenia Castro, Miguel Pinedo, Craig A. Field, Lin Tran, Ty S. Schepis

**Affiliations:** 1 Department of Psychology, Texas State University, San Marcos, TX USA; 2 Alcohol Research Group, Public Health Institute, Emeryville, CA USA; 3 Steve Hicks School of Social Work, The University of Texas at Austin, Austin, TX USA; 4 Department of Kinesiology and Health Education, The University of Texas at Austin, Austin, TX USA; 5 Department of Psychology, The University of Texas at El Paso, El Paso, TX USA

**Keywords:** Alcohol, AUD, Race, Ethnicity, Sex, Adult

## Abstract

**Purpose:**

Population-based studies of alcohol-related trends typically collapse across sex while examining race and/or age, limiting understanding of shifts in alcohol involvement at the intersection of sex, race, and age. Therefore, this study evaluated population-level trends in alcohol use and alcohol use disorder (AUD) symptoms as disaggregated within Hispanic, Black, and White female and male U.S. early and middle adults.

**Methods:**

Data were from years 2002 to 2019 of the National Survey on Drug Use and Health, Participants were 18 to 64, Hispanic, Black, or White, and consumed any alcohol. Annualized linear change estimates were computed to assess trends in past-month drinking days and AUD symptoms. Between groups analyses were also conducted to examine (a) sex differences within ethnoracial identity and (b) ethnoracial differences within sex. All analyses were further stratified across early (age 18 to 29) and middle adults (age 30 to 64).

**Results:**

Number of drinking days increased only among Black early adult females and Black middle adult females and decreased for all males except for Black middle adults, with the strongest decrease for Black early adult males. AUD symptoms decreased for all early adult males, and most strongly among Black males. Among middle adults, AUD symptoms decreased only among Hispanic males and increased among White males and females.

**Conclusions and relevance:**

National trends in alcohol use and AUD symptoms are distinct across subpopulations at the intersection of sex, race, and age. Continued disaggregated analyses across heterogeneous U.S. subpopulations are needed to better inform clinical care.

## Introduction

Problematic alcohol use contributes to numerous adverse consequences such as job loss, financial troubles, and an increase in emergency room visits [1]. Alcohol-related mortality among U.S. adults is increasing [2, 3], and this trend is projected to continue [4]. A meta-analysis of U.S. national datasets indicates the prevalence of alcohol use and binge drinking increased by 4.5% and 10.8%, respectively, from 2000 to 2015 [5]. Another study observed a 4.2% increase in alcohol use disorder (AUD) from 2002 to 2014 [5]. Research also shows differential trends in alcohol use and AUD across ethnoracial identity such that the rates of increase in alcohol use [3, 5] and AUD [3] were larger among Black and Hispanic adults compared with non-Hispanic White (White) adults. Further, evidence suggests that between-group differences in AUD across ethnoracial identity are conditional upon life stage (i.e., early vs. middle/late adulthood) [6].

Sex differences in alcohol trends and AUD are documented in population-based studies. Though women have historically consumed less alcohol and report less AUD than men [6–8], there is robust evidence that the gender gap in alcohol use is narrowing [5, 7, 9–11]. For instance, research shows significant increases in alcohol use and prevalence of AUD among U.S. women, but not men [5, 7]. Thus, alcohol use and AUD profiles are changing in complex ways that vary by sex, ethnoracial identity, and their intersection.

Past research illuminating ethnoracial and sex differences in alcohol-related trends typically does not disaggregate ethnoracial identity from sex. That is, group comparisons are typically reported for ethnoracial identity while overlooking sex or are reported for sex while not considering ethnoracial identity [5–8, 12, 13]. This hinders the application of extant knowledge by researchers and practitioners focused on and/or working with members of specific populations (e.g., Black males, Hispanic females). Importantly, studies indicate that alcohol-related harms are increasing among some populations more than others. For instance, alcohol-related mortality is increasing more strongly among White and Black female adults than their male counterparts, but the converse is true for Hispanic adults [14]. As such, a disaggregated analysis of population-based trends in alcohol use and AUD symptoms across U.S. female and male adults from prominent ethnoracial groups while also stratifying across age group can inform precision medicine [15] efforts for screening, prevention, and treatment tailored toward individuals within the heterogeneous U.S. population.

### Current study

The aim of this study was to explore population-based trends in alcohol use and AUD symptoms from 2002 to 2019 among Hispanic, Black, and White adults in the U.S., disaggregating across sex and ethnoracial identity. The focus of this study on AUD symptoms rather than prevalence of discrete diagnostic AUD categories is based on evidence that subthreshold levels of AUD are associated with heavier drinking and increased risk for later AUD [16, 17]. Analyses were conducted separately for early adults (ages 18–29) and middle adults (ages 30–64) and guided by the following research questions (RQs):

#### RQ1

What were the individual trends in alcohol use and AUD symptoms among Hispanic, Black, and White early and middle adults in the U.S.?

#### RQ2

Within each ethnoracial group and each age group, what were the sex differences in alcohol use and AUD symptoms?

#### RQ3

Within each sex and each age group, what were the ethnoracial differences in alcohol use and AUD symptoms?

For RQ2 and RQ3, we were interested in measuring both overall differences (i.e., collapsed across all study years) and differences in trends over time.

## Method

### Data source

The National Survey on Drug Use and Health (NSDUH), years 2002 to 2019. Data from 2020 and beyond were not included due to COVID-related changes in the NSDUH that precluded comparisons with prior years [18]. The NSDUH served as the data source for several reasons. First, data are collected annually, with sufficient samples to afford meaningful subgroup comparisons across Hispanic, Black, and White female and male early and middle adults. Next, unlike other large annual surveys [19], the NSDUH collects data on AUD symptoms. Further, a meta-analysis of alcohol use measured in national surveys indicated that NSDUH estimates were the most closely aligned with overall meta-analytic estimates [5]. The NSDUH implements an independent, multistage area probability sampling strategy with person-level weights for nationally representative estimates of the U.S. population. See the Methodological Resource Book [20] for more information.

### Participants

Hispanic, Black, and White adults aged 18 to 64 who reported any alcohol consumption were drawn from NSDUH years 2002 to 2019 (*N* = 519,314). Age groups were formed based on NSDUH available categories and feasibility of statistical analysis given power limitations of further disaggregation. The NSDUH categorized age in years as: 18; 19; 20; 21; 22 or 23; 24 or 25; between 26 and 29; between 30 and 34; between 35 and 49; between 50 and 64. The weighted percentages with standard errors (SE) of the total sample were as follows: Among early adults aged 18–29, 10.3% (0.09) were Hispanic females, 11.5% (0.12) were Hispanic males, 7.8% (0.08) were Black females, 7.0% (0.08) were Black males, 31.6% (0.13) were White females, and 31.8% (0.16) were White males. Among middle adults aged 30 to 64, 7.7% (0.08) were Hispanic females, 7.8% (0.08) were Hispanic males, 6.9% (0.08) were Black females, 5.7% (0.07) were Black males, 36.4% (0.15) were White females, and 35.4% (0.14) were White males.

### Measures

Categories for *sex* and *ethnoracial identity* were derived from the NSDUH codebook [18]: Black females, Black males, Hispanic females, Hispanic males, White females, and White males. The NSDUH has an additional composite category for “Male or Female, Other Races,” which was omitted from analyses due to small sample sizes. Alcohol use was assessed as *past-month drinking days* in which participants self-reported the number of days they consumed any alcohol within the past month, with a possible range of 0 to 30 days. For *AUD symptoms*, participants reported on 10 of 11 AUD criteria per the DSM-IV. The NSDUH does not measure the DSM-5 criterion of craving before 2020 [18]. For each criterion, responses were coded as 1 (criterion met) or 0 (criterion not met); these were summed for a total score. Covariates included ordinal *age* (10-point scale, from “18 years old” to “Between 50 and 64 years old”), *marital status* (1 = married/0 = not married), *in-school status* (1 = currently student/0 = not current student), and *highest level of education* (range from 1 = less than high school to 4 = college graduate). For descriptive purposes, we also report on *current student status* and *income* (range from 1 = less than $20,000 to 4 = $75,000 or more).

### Analytic approach

Analyses were conducted in Stata 17.0 and incorporated NSDUH-provided complex survey variables and adjusted person-level weights. Robust variances with adjusted degrees of freedom were computed with the Taylor series approximation. For RQ1, we used negative binomial regressions within each of the 12 subgroups (i.e., Hispanic females and males, Black females and males, and White females and males stratified across early adults and middle adults). Annualized linear change (ALC) was assessed via a margins command following each regression. For RQ2 and RQ3, we conducted negative binomial regressions to examine between-group differences in overall estimates of the alcohol variables (combined across years) and differences in trends of the alcohol variables across years. Rather than designate a single reference group to which all other subgroups were compared, all possible pairwise comparisons across the subgroups within age groups were assessed, yielding 15 pairwise comparisons per outcome within each age group. Therefore, we applied a Benjamini-Hochberg alpha correction with a 5% false discovery rate [21] to identify significant effects.

**Table 1:** Stratified sample characteristics

	Early Adults (18 to 29)					
	Hispanic Females	Hispanic Males	Black Females	Black Males	White Females	White Males
Married (Ref: Not Married)	27.45%(0.43)	19.91%(0.38)	10.95%(0.29)	9.58%(0.34)	27.13%(0.23)	17.88%(0.21)
Current Student (Ref: Not Current Student)	33.63%(0.40)	28.38%(0.44)	39.04%(0.54)	34.08%(0.48)	37.04%(0.26)	34.63%(0.27)
Highest Level of Education						
Less than high school	23.13%(0.37)	29.34%(0.47)	14.87%(0.33)	19.91%(0.41)	10.32%(0.14)	13.98%(0.15)
High school graduate	33.60%(0.40)	36.18%(0.44)	35.75%(0.40)	39.39%(0.47)	26.47%(0.20)	31.37%(0.24)
Some college	32.29%(0.39)	26.43%(0.52)	36.13%(0.46)	30.07%(0.49)	36.29%(0.24)	33.20%(0.22)
College graduate	10.97%(31.84)	8.05%(0.32)	13.25%(0.36)	10.63%(0.35)	26.91%(0.25)	21.45%(0.22)
Income						
Less than $20,000	31.49%(< 0.01)	24.77%(< 0.01)	41.42%(0.01)	30.96%(< 0.01)	24.57%(< 0.01)	21.80%(< 0.01)
$20,000 to $49,000	41.39%(< 0.01)	43.27%(< 0.01)	37.64%(< 0.01)	40.56%(0.01)	34.14%(< 0.01)	32.75%(< 0.01)
$50,000 to $74,999	12.87%(< 0.01)	14.74%(< 0.01)	10.04%(< 0.01)	13.93%(< 0.01)	16.92%(< 0.01)	17.10%(< 0.01)
$75,000 or more	14.25%(< 0.01)	17.22%(< 0.01)	10.91%(< 0.01)	14.56%(< 0.01)	24.37%(< 0.01)	28.35%(< 0.01)
Past Month Drinking Days	3.02(0.04)	4.69(0.06)	3.30(0.05)	5.00(0.09)	4.66(0.03)	6.99(0.04)
AUD Symptoms	0.99(0.01)	1.53(0.02)	0.98(0.01)	1.37(0.02)	1.22(0.01)	1.55(0.01)

	Middle Adults (30 to 64)					
	Hispanic Females	Hispanic Males	Black Females	Black Males	White Females	White Males
Married (Ref: Not Married)	60.57%(0.46)	64.49%(0.42)	36.45%(0.45)	48.72%(0.63)	67.24%(0.23)	68.08%(0.23)
Current Student (Ref: Not Current Student)	5.02%(0.20)	3.85%(0.20)	9.25%(0.29)	5.75%(0.27)	4.54%(0.09)	3.17%(0.08)
Highest Level of Education						
Less than high school	35.15%(0.49)	36.75%(0.56)	13.68%(0.34)	16.93%(0.47)	6.92%(0.11)	8.77%(0.12)
High school graduate	24.63%(0.43)	25.72%(0.42)	31.78%(0.54)	36.34%(0.55)	26.94%(0.19)	29.01%(0.22)
Some college	22.65%(0.34)	21.41%(0.45)	31.80%(0.49)	27.56%(0.45)	29.67%(0.19)	25.56%(0.18)
College graduate	17.56%(0.37)	16.12%(0.43)	22.74%(0.47)	19.17%(0.47)	36.47%(0.24	36.66%(0.24)
Income						
Less than $20,000	25.93%(< 0.01)	19.59%(< 0.01)	30.77%(< 0.01)	23.96%(< 0.01)	10.84%(< 0.01)	9.05%(< 0.01)
$20,000 to $49,000	39.82%(< 0.01)	41.44%(0.01)	36.18%(< 0.01)	35.01%(0.01)	26.89%(< 0.01)	24.42%(< 0.01)
$50,000 to $74,999	14.37%(< 0.01)	15.07%(< 0.01)	14.43%(< 0.01)	16.50%(< 0.01)	19.58%(< 0.01)	19.10%(< 0.01)
$75,000 or more	19.89%(< 0.01)	23.90%(0.01)	18.63%(< 0.01)	24.53%(0.01)	42.69%(< 0.01)	47.43%(< 0.01)
Past Month Drinking Days	2.50(0.06)	4.48(0.07)	3.12(0.06)	5.53(0.09)	4.92(0.04)	7.42(0.05)
AUD Symptoms	0.49(0.01)	1.13(0.02)	0.66(0.01)	1.16(0.02)	0.62(0.01)	0.92(0.01)

Note. Percentages(SE) are presented for categorical data and Means(SE) are presented for continuous data

## Results

See Table 1 for stratified sample characteristics and descriptive statistics.

### RQ1: Within-group trends

#### Past-Month drinking days (Table 2)

##### Early adults

Past-month drinking days among females significantly increased only among Black females (*p* < 0.001). Conversely, past-month drinking days among males from all groups significantly decreased. The decrease was largest for Black males (*p* < 0.001) and smallest for Hispanic males (*p* < 0.001).

##### Middle adults

As with early adults, past-month drinking days among middle adult females significantly increased only among Black females (*p* = 0.001). Among middle adult males, past-month drinking days decreased among Hispanic (*p* = 0.008) and White males (*p* < 0.001).

#### AUD symptoms (Table 3)

##### Early adults

Among females, there were no significant changes in AUD symptoms. Among males, AUD symptoms significantly decreased within all three groups. The largest decrease was for Black males (*p* < 0.001) and the smallest was for White males (*p* < 0.001).

##### Middle adults

The only significant trend in AUD symptoms among females was an increase among White middle adults (*p* < 0.001). Among males, AUD symptoms decreased for Hispanic middle adults (*p* = 0.04). Conversely, AUD symptoms increased among White males (*p* < 0.001).

### RQ2 & RQ3 tests of between-groups differences

**Table 2:** Past-month drinking days

Hispanic Adults									Black Adults								White Adults							
Year	Aged 18 to 29				Aged 30 to 64				Aged 18 to 29				Aged 30 to 64				Aged 18 to 29				Aged 30 to 64			
	Females		Males		Females		Males		Females		Males		Females		Males		Females		Males		Females		Males	
	M	SE	M	SE	M	SE	M	SE	M	SE	M	SE	M	SE	M	SE	M	SE	M	SE	M	SE	M	SE
2002	2.58	0.24	4.28	0.23	2.45	0.39	5.15	0.38	2.49	0.19	5.83	0.43	2.72	0.30	6.18	0.36	4.15	0.10	7.00	0.13	4.86	0.14	7.85	0.20
2003	2.57	0.16	5.29	0.29	2.09	0.21	4.41	0.30	2.85	0.25	5.37	0.32	2.51	0.22	5.85	0.48	4.24	0.14	7.21	0.18	4.87	0.16	7.59	0.19
2004	2.66	0.17	5.00	0.31	2.27	0.24	4.74	0.35	2.87	0.19	5.41	0.33	2.64	0.28	5.38	0.34	4.23	0.08	7.31	0.16	5.01	0.19	7.67	0.18
2005	2.47	0.14	4.95	0.30	2.80	0.36	4.44	0.34	2.65	0.19	5.80	0.53	3.25	0.36	5.47	0.43	4.42	0.11	7.25	0.18	4.88	0.16	7.48	0.16
2006	2.64	2.00	4.98	0.47	2.43	0.21	5.18	0.30	2.61	0.19	5.93	0.40	2.79	0.23	6.09	0.46	4.58	0.13	7.48	0.15	4.72	0.12	7.26	0.19
2007	2.80	0.20	4.93	0.26	2.54	0.19	4.24	0.29	2.99	0.19	5.21	0.41	3.07	0.27	5.08	0.42	4.54	0.12	7.39	0.16	4.44	0.14	7.65	0.20
2008	2.82	0.17	5.19	0.27	2.79	0.23	5.11	0.45	3.28	0.18	5.77	0.45	2.78	0.25	6.59	0.61	4.78	0.14	7.34	0.17	4.83	0.15	7.36	0.21
2009	3.23	0.22	4.98	0.30	2.00	0.19	4.60	0.31	3.59	0.24	5.03	0.27	2.83	0.21	5.35	0.42	4.70	0.12	7.36	0.15	4.93	0.16	7.4	0.22
2010	3.05	0.22	4.41	0.29	2.52	0.26	4.21	0.33	3.26	0.18	5.03	0.42	2.76	0.23	4.85	0.36	4.49	0.10	7.09	0.16	4.96	0.17	7.52	0.16
2011	3.16	0.26	4.70	0.31	2.34	0.24	4.60	0.34	3.32	0.18	5.53	0.53	3.47	0.27	5.03	0.32	4.81	0.12	6.92	0.16	5.1	0.17	7.16	0.19
2012	2.99	0.18	4.52	0.26	2.63	0.28	4.29	0.39	3.57	0.22	4.63	0.30	3.33	0.26	5.4	0.49	4.82	0.11	7.05	0.17	5.08	0.14	7.63	0.20
2013	3.15	0.16	4.39	0.26	2.74	0.25	4.48	0.38	3.59	0.26	4.93	0.35	3.01	0.26	5.07	0.40	5.02	0.17	6.85	0.17	5.11	0.16	7.55	0.20
2014	3.28	0.16	4.58	0.25	2.71	0.21	4.74	0.30	3.96	0.24	4.82	0.24	3.58	0.22	5.39	0.30	5.00	0.12	6.85	0.19	5.09	0.11	7.66	0.15
2015	3.35	0.17	4.21	0.18	2.72	0.29	4.27	0.20	3.47	0.21	4.35	0.27	3.56	0.21	5.54	0.27	4.75	0.13	6.95	0.16	4.96	0.12	7.34	0.17
2016	3.24	0.18	5.12	0.41	2.24	0.18	4.14	0.26	3.54	0.20	4.25	0.19	3.28	0.21	6.08	0.38	4.94	0.13	6.58	0.14	5.03	0.16	7.26	0.15
2017	3.16	0.21	4.51	0.24	2.57	0.17	4.56	0.31	3.62	0.20	4.25	0.26	3.48	0.25	5.8	0.33	5.03	0.15	6.40	0.18	4.86	0.11	7.01	0.18
2018	3.46	0.21	4.25	0.17	2.51	0.18	4.03	0.21	3.58	0.34	4.24	0.31	3.62	0.30	5.42	0.32	4.75	0.14	6.30	0.16	4.94	0.14	7.11	0.18
2019	3.07	0.15	4.56	0.23	2.58	0.17	4.00	0.19	3.96	0.23	3.94	0.28	3.29	0.22	5.04	0.26	4.76	0.13	6.15	0.16	4.79	0.17	7.05	0.15
ALC [95% CI]	0.0169 [-0.0011 to 0.0349]		− 0.0771*** [-0.1001 to -0.0542]		0.0106 [-0.0131 to 0.0344]		-0.0413** [-0.0690 to -0.0135		0.0605*** [0.0399 to 0.0812]		-0.1329*** [-0.1654 to -0.1004]		0.0537*** [0.0246 to 0.0829]		-0.0242 [-0.0605 to 0.0120]		0.0041 [-0.0074 to 0.0155]		-0.1079*** [-0.1229 to -0.0929]		0.0009 [-0.0047 to 0.0230]		-0.0306*** [-0.0466 to -0.0146]	

Note. ALC = Annualized Linear Change; CI = Confidence Interval of ALC; ***p < 0.001, **p < 0.01, *p < 0.050

**Table 3:** Predicted value of AUD symptoms across year

Hispanic Adults										Black Adults											White Adults											
Year	Aged 18 to 29				Aged 30 to 64					Aged 18 to 29					Aged 30 to 64						Aged 18 to 29							Aged 30 to 64				
	Females		Males		Females		Males			Females		Males			Females			Males			Females			Males			Females				Males	
	M	SE	M	SE	M	SE	M	SE	M		SE	M	SE	M		SE	M		SE	M		SE	M		SE	M			SE	M		SE
2002	1.65	0.10	2.31	0.13	0.96	0.10	1.70	0.11	1.52		0.10	2.47	0.19	1.32		0.10	1.96		0.12	1.57		0.04	2.11		0.04	0.85			0.04	1.12		0.03
2003	1.65	0.10	2.51	0.15	0.80	0.09	1.58	0.09	1.51		0.08	2.30	0.12	1.10		0.09	1.76		0.13	1.66		0.04	2.07		0.04	0.87			0.03	1.09		0.03
2004	1.68	0.09	2.6	0.18	1.04	0.09	1.77	0.12	1.61		0.12	2.38	0.12	1.41		0.10	1.75		0.14	1.69		0.04	2.13		0.04	0.97			0.04	1.21		0.04
2005	1.85	0.10	2.18	0.10	1.06	0.08	1.81	0.14	1.57		0.10	2.33	0.15	1.32		0.10	1.68		0.11	1.8		0.05	2.09		0.05	0.88			0.03	1.16		0.04
2006	1.89	0.10	2.43	0.10	0.99	0.10	1.67	0.14	1.60		0.11	2.17	0.09	1.15		0.08	1.69		0.12	1.69		0.05	2.15		0.05	0.89			0.03	1.19		0.04
2007	1.53	0.09	2.27	0.10	1.06	0.12	1.72	0.14	1.66		0.10	2.19	0.11	1.31		0.08	2.10		0.13	1.73		0.04	2.10		0.05	0.9			0.03	1.29		0.05
2008	1.93	0.10	2.49	0.14	0.99	0.08	1.67	0.14	1.72		0.09	2.31	0.12	1.28		0.08	1.91		0.15	1.83		0.04	2.04		0.04	1.00			0.04	1.25		0.06
2009	1.60	0.09	2.38	0.10	1.13	0.12	1.66	0.12	1.68		0.09	2.29	0.12	1.12		0.07	2.01		0.17	1.69		0.05	2.05		0.05	0.97			0.03	1.24		0.04
2010	1.91	0.12	2.34	0.10	0.96	0.10	1.58	0.11	1.68		0.13	2.17	0.12	1.26		0.08	1.70		0.10	1.70		0.04	2.02		0.04	0.90			0.04	1.20		0.03
2011	1.85	0.14	2.17	0.15	1.13	0.11	1.68	0.11	1.52		0.07	2.05	0.11	1.27		0.09	1.77		0.11	1.66		0.05	1.93		0.04	0.90			0.03	1.20		0.04
2012	1.86	0.14	2.29	0.09	0.99	0.09	1.71	0.12	1.78		0.08	2.13	0.12	1.17		0.09	1.80		0.11	1.61		0.05	2.05		0.05	0.92			0.04	1.25		0.04
2013	1.81	0.11	2.11	0.10	1.08	0.10	1.82	0.12	1.55		0.09	2.02	0.12	1.27		0.09	1.90		0.10	1.60		0.05	1.85		0.04	0.92			0.04	1.27		0.04
2014	1.73	0.09	2.19	0.10	1.04	0.06	1.58	0.09	1.50		0.07	2.11	0.11	1.35		0.10	1.89		0.09	1.58		0.04	1.94		0.05	0.95			0.02	1.21		0.03
2015	1.90	0.11	2.21	0.09	1.02	0.08	1.76	0.08	1.51		0.06	1.93	0.09	1.23		0.07	1.76		0.09	1.55		0.04	1.86		0.05	1.06			0.04	1.36		0.03
2016	1.73	0.08	1.98	0.09	1.02	0.06	1.65	0.08	1.64		0.08	2.09	0.08	1.28		0.08	1.98		0.13	1.70		0.04	1.79		0.05	1.03			0.04	1.30		0.04
2017	1.59	0.09	2.04	0.10	1.15	0.08	1.57	0.09	1.47		0.06	1.85	0.10	1.32		0.08	1.64		0.09	1.69		0.05	1.88		0.05	1.12			0.03	1.39		0.03
2018	1.76	0.11	2.08	0.11	1.00	0.07	1.58	0.08	1.71		0.09	1.91	0.09	1.33		0.09	1.60		0.09	1.67		0.05	1.92		0.05	1.08			0.03	1.39		0.04
2019	1.81	0.08	2.23	0.12	1.07	0.06	1.47	0.08	1.77		0.08	1.75	0.09	1.46		0.08	1.83		0.12	1.64		0.04	1.9		0.04	1.12			0.04	1.44		0.04
ALC [95% CI]	0.0022 [-0.0062 to 0.0106]		-0.0234*** [-0.0347 to -0.0120]		0.0056 [-0.0017 to 0.0129]		-0.0086* [-0.0169 to -0.0002]			0.0027 [-0.0055 to 0.0109]		-0.0336*** [-0.0454 to -0.0218]			0.0060 [-0.0027 to 0.0148]			-0.0047 [-0.0144 to 0.0051]			-0.0038 [-0.0081 to 0.0006]			-0.0180*** [-0.0222 to -0.0137]			0.0237*** [0.0100 to 0.0158]				0.0150*** [0.0117 to 0.0182]	

Note. ALC = Annualized Linear Change; CI = Confidence Interval of ALC; ***p < 0.001, **p < 0.01, *p < 0.050

#### Past-Month drinking days (Table 4)

##### Early adults

*Within all ethnoracial groups*, females reported fewer overall (i.e., collapsed across NSDUH year) drinking days than males (*p*s < 0.001). The increasing trend for past-month drinking days across years for females was different than the decreasing trend for males within the same ethnoracial group (*p*s < 0.001). *Within sex* analyses indicated that White females reported more overall past-month drinking days than Hispanic and Black females (*p*s < 0.001). The increasing trend of past-month drinking days across years was different for Black females compared to White females (*p* < 0.001). Hispanic and Black males reported fewer overall past-month drinking days than White males (*p*s < 0.001). The decreasing trend of past-month drinking days was stronger for Black males compared to Hispanic males (*p* = 0.02) and White males (*p* = 0.002).

##### Middle adults

Many findings for middle adults were similar to findings for early adults. *Within each ethnoracial group*, females reported fewer overall drinking days than males (*p*s < 0.001). In addition, the decrease across years in past-month drinking days among males was different than the increase among females (Hispanic middle adults: *p* = 0.001, Black and White middle adults: *p* < 0.001), though we note the nonsignificant ALC estimates reported for Hispanic and White middle adult females and Black middle adult males reported earlier. *Within sex*, Hispanic females reported fewer overall past-month drinking days than Black females and White females, while Black females reported significantly fewer drinking days than White females (*p*s < 0.001). Hispanic males reported fewer overall past-month drinking days than Black males, who reported fewer past-month drinking days than White males (*p*s < 0.001). There were no significant differences in the trend over NSDUH years of past-month drinking days across ethnoracial group within sex.

**Table 4:** Coefficients and confidence intervals from pairwise analysis of Past-Month drinking days

Incidence Rate Ratio for Past-Month Drinking Days, Ages 18 to 29																
Main Effect of Subgroup								Interaction Effect of Subgroup*Year								
		Hispanic Females	Hispanic Males	Black Females	Black Males	White Females	White Males			Hispanic Females	Hispanic Males	Black Females	Black Males	White Females	White Males	
Reference Group	Hispanic Females	-	1.60*** [1.54,** 1.65]**	1.03 [0.98, 1.07]	1.60*** [1.52,** 1.67]**	1.43*** [1.38,** 1.47]**	2.18*** [2.12,** 2.25]**	Reference Group	Hispanic Females	-	0.97*** [0.97,** 0.98]**	1.01 [1.00, 1.02]	0.96*** [0.96,** 0.97]**	0.99 [0.99, 1.00]	0.98*** [0.97,** 0.98]**	
	Hispanic Males		-	0.64*** [0.62,** 0.67]**	1.00 [0.96, 1.05]	0.89*** [0.87,** 0.92]**	1.37*** [1.33,** 1.40]**		Hispanic Males		-	1.03*** [1.03,** 1.04]**	0.99** [0.98,** 1.00]**	1.02*** [1.01,** 1.02]**	1.00 [1.00, 1.00]	
	Black Females			-	1.55*** [1.48,** 1.63]**	1.39*** [1.34,** 1.44]**	2.12*** [2.05,** 2.20]**		Black Females			-	0.96*** [0.95,** 0.97]**	0.99*** [0.98,** 0.99]**	0.97*** [0.96,** 0.98]**	
	Black Males				-	0.89*** [0.86,** 0.93]**	1.37*** [1.31,** 1.42]**		Black Males				-	1.03*** [1.02,** 1.04]**	1.01*** [1.00,** 1.02]**	
	White Females					-	1.53*** [1.50,** 1.55]**		White Females					-	0.98*** [0.98,** 0.98]**	
	White Males						-		White Males						-	

Incidence Rate Ratio for Past-Month Drinking Days, Ages 30 to 64																
Main Effect of Subgroup								Interaction Effect of Subgroup*Year								
		Hispanic Females	Hispanic Males	Black Females	Black Males	White Females	White Males			Hispanic Females	Hispanic Males	Black Females	Black Males	White Females	White Males	
Reference Group	Hispanic Females	-	1.91*** [1.81,** 2.03]**	1.28*** [1.20,** 1.36]**	2.36*** [2.22,** 2.50]**	1.79*** [1.70,** 1.88]**	2.75*** [2.62,** 2.89]**	Reference Group	Hispanic Females	-	0.98** [0.97,** 0.99]**	1.00 [0.99, 1.02]	0.98** [0.97,** 0.99]**	1.00 [0.99, 1.01]	0.99** [0.98,** 1.00]**	
	Hispanic Males		-	0.67*** [0.63,** 0.70]**	1.23*** [1.18,** 1.29]**	0.93** [0.90,** 0.97]**	1.44*** [1.39,** 1.49]**		Hispanic Males		-	1.02*** [1.01,** 1.03]**	1.00 [0.99, 1.01]	1.01*** [1.01,** 1.02]**	1.01 [1.00, 1.01]	
	Black Females			-	1.84*** [1.74,** 1.95]**	1.40*** [1.34,** 1.46]**	2.15*** [2.06,** 2.25]**		Black Females			-	0.98*** [0.97,** 0.99]**	0.99 [0.98, 1.00]	0.98** [0.97,** 0.99]**	
	Black Males				-	0.76*** [0.73,** 0.79]**	1.17*** [1.13,** 1.21]**		Black Males				-	1.01*** [1.01,** 1.02]**	1.00 [1.00, 1.01]	
	White Females					-	1.54*** [1.51,** 1.57]**		White Females					-	0.99*** [0.99,** 0.99]**	
	White Males						-		White Males						-	

Note. Results presented as coefficient [95% confidence interval]; Reference group notated along rows. ***P < 0.001, **P < 0.01, *P < 0.05; Benjamini-Hochberg alpha corrected significant effects highlighted in bold. All analyses adjusted for ordinal age, marital status, highest level of education, and income

#### AUD symptoms (Table 5)

##### Early adults

*Within each ethnoracial group*, females reported lower overall AUD symptoms than males (*p*s < 0.001). The decreasing trend in AUD symptoms among males was significantly different than the trend for females (*p*s < 0.001), though we note the nonsignificant ALC estimates among all females reported earlier. *Within sex*, Hispanic females reported greater overall AUD symptoms than Black females and White females (*p*s < 0.001). Hispanic males also reported greater overall AUD symptoms than Black males and White males while Black males reported greater overall AUD symptoms than White males (*p*s < 0.001). The effect of year on AUD symptoms was not significantly different across ethnoracial identity within sex for any group.

##### Middle adults

Similar to early adults, *within each ethnoracial group*, female middle adults reported lower overall AUD symptoms than males (*p*s < 0.001). Unlike the early adult sample, there were no significant differences in trends of AUD symptoms across sex within each ethnoracial group following alpha correction. *Within sex*, Black females reported significantly higher overall AUD symptoms than Hispanic (*p* = 0.001) and White females (*p* < 0.001), while Hispanic females reported significantly higher overall AUD symptoms than White females (*p* < 0.001). However, there were no statistically significant effects of year on AUD symptoms across ethnoracial group among females after alpha correction. Hispanic and Black males reported higher overall AUD symptoms than White males (*p*s < 0.001). However, in terms of differences in trends, the increase in AUD symptoms among White males was significantly different than the decreasing trend among Hispanic and Black males (*p*s < 0.001), though we note the ALC estimate reported earlier for Black middle adult males was nonsignificant.

## Discussion

This study examined within- and between-group trends in alcohol use and AUD symptoms among Hispanic, Black, and White female and male adults in the U.S., also disaggregating across early adults and middle adults. Findings from the current study highlight the importance of disaggregating analyses by subgroups for a more nuanced understanding of shifts in alcohol use and AUD symptoms among U.S. adults. That is, these results present more granular and thus useful information about for *whom* and to *what extent* the prevalence of alcohol use and AUD symptoms is shifting.

### RQ1 findings: trends in alcohol use and AUD symptoms within sex, ethnoracial group, and age group

As alcohol use was measured with past-month drinking days, results pertaining to this variable may be interpreted as a measure of frequency of drinking. Contrary to literature indicating rising alcohol use among U.S. adult females [5, 7], only Black females significantly increased their frequency of consumption. Consistent with literature [10], frequency of drinking significantly declined among males of all ethnoracial and age groups, with a single exception for Black middle adult males. Black individuals in the U.S. have historically consumed alcohol less frequently than other groups [22], potentially making increases more apparent or reductions less apparent than for groups who drink more frequently.

**Table 5:** Coefficients and confidence intervals from pairwise analysis of AUD symptoms

Incidence Rate Ratio for AUD symptoms, Ages 18 to 29															
Main Effect of Subgroup								Interaction Effect of Subgroup*Year							
		Hispanic Females	Hispanic Males	Black Females	Black Males	White Females	White Males			Hispanic Females	Hispanic Males	Black Females	Black Males	White Females	White Males
Reference Group	Hispanic Females	-	1.17*** [1.13,** 1.21]**	0.86*** [0.83,** 0.89]`**	1.05** [1.01,** 1.09]**	0.88*** [0.85,** 0.90]**	0.92*** [0.90,** 0.95]**	Reference Group	Hispanic Females	-	0.99** [0.98,** 0.99]**	1.00 [0.99, 1.01]	0.99*** [0.98,** 0.99]**	1.00 [0.99, 1.00]	0.99*** [0.98,** 0.99]**
	Hispanic Males		-	0.74*** [0.71,** 0.77]**	0.90*** [0.86,** 0.93]**	0.75*** [0.73,** 0.77]**	0.79*** [0.77,** 0.81]**		Hispanic Males		-	1.01** [1.01,** 1.02]**	1.00 [0.99, 1.01]	1.01** [1.00,** 1.01]**	1.00 [1.00, 1.01]
	Black Females			-	1.22*** [1.17,** 1.27]**	1.02 [0.99, 1.05]	1.07*** [1.04,** 1.10]**		Black Females			-	0.98*** [0.98,** 0.99]**	1.00 [0.99, 1.00]	0.99*** [0.98,** 1.00]**
	Black Males				-	0.84*** [0.81,** 0.86]**	0.88*** [0.85,** 0.91]**		Black Males				-	1.01*** [1.01,** 1.02]**	1.01 [1.00, 1.01]
	White Females					-	1.05*** [1.03,** 1.07]**		White Females					-	0.99*** [0.99,** 1.00]**
	White Males						-		White Males						-

Incidence Rate Ratio for AUD symptoms, Ages 30 to 64															
Main Effect of Subgroup								Interaction Effect of Subgroup*Year							
		Hispanic Females	Hispanic Males	Black Females	Black Males	White Females	White Males			Hispanic Females	Hispanic Males	Black Females	Black Males	White Females	White Males
Reference Group	Hispanic Females	-	1.42*** [1.35,** 1.49]**	1.08** [1.03,** 1.14]**	1.40*** [1.33,** 1.48]**	0.90*** [0.87,** 0.93]**	1.01 [0.97, 1.05]	Reference Group	Hispanic Females	-	0.99* [0.98,** 1.00]**	1.00 [0.99, 1.01]	0.99 [0.98, 1.00]	1.01 [1.00, 1.02]	1.00 [1.00, 1.01]
	Hispanic Males		-	0.73*** [0.70,** 0.77]**	0.97 [0.93, 1.02]	0.60*** [0.58,** 0.62]**	0.68*** [0.66,** 0.71]**		Hispanic Males		-	1.01* [1.00, 1.02]	1.00 [1.00, 1.01]	1.02*** [1.01,** 1.02]**	1.02*** [1.01,** 1.02]**
	Black Females			-	1.30*** [1.24,** 1.36]**	0.83*** [0.80,** 0.86]**	0.93*** [0.90,** 0.97]**		Black Females			-	0.99 [0.99, 1.00]	1.01* [1.00, 1.02]	1.01 [1.00, 1.01]
	Black Males				-	0.64*** [0.61,** 0.67]**	0.72*** [0.69,** 0.75]**		Black Males				-	1.02*** [1.01,** 1.02]**	1.01*** [1.01,** 1.02]**
	White Females					-	1.12*** [1.10,** 1.15]**		White Females					-	1.00 [0.99, 1.00]
	White Males						-		White Males						-

Note. Results presented as coefficient [95% confidence interval]; Reference group notated along rows. ***p < 0.001, **p < 0.01, *p < 0.05; Benjamini-Hochberg alpha corrected significant effects highlighted in bold. All analyses adjusted for number of past-month drinking days, ordinal age, marital status, highest level of education, and income

AUD symptoms among White middle adult females increased from 2002 to 2019 but did not significantly change for any other female group. Trends in AUD symptoms across groups of males were more varied. Among early adults, AUD symptoms decreased for all males. For middle adults, AUD symptoms decreased only for Hispanic males and increased for White males. This contrasts prior research indicating stronger increases in the prevalence of AUD among Hispanic and Black adults compared to White adults when collapsing across age and sex [12]. These findings underscore the utility of disaggregating analyses across ethnoracial identity, sex, and age, as well as measuring AUD symptoms rather than AUD prevalence.

### RQ2 & RQ3 findings: sex and ethnoracial group differences in trends in alcohol use and AUD symptoms within age group

Between-groups analyses of trends in alcohol use were largely consistent with previous research reporting a narrowing of the gender gap in drinking. That is, the downward trends in frequency of alcohol consumption among males were significantly different than the upward – or null – trends among females. Analyses of AUD symptoms further indicated that the gender gap in drinking is narrowing among early adults within each racial and ethnic group, predominantly driven by the reductions among males. Scholars surmise that rising rates of drinking and AUD among women are due in part to shifting roles, such as having a more prominent presence in the workforce and delaying or bypassing parenthood [23] There is also a growing trend on social media promoting alcohol use among women, such as the “wine mom culture,” [24] which is described in research as a predominantly White female phenomenon [25].

Although research on masculinity and alcohol use historically characterizes excessive drinking as a way men can express masculinity [26, 27], some research highlights positive aspects of masculinity that may protect against risky alcohol use [30–32]. Relevant to this study’s findings on AUD symptoms among Hispanic middle adult males, research on the protective aspects of masculinity in relation to drinking has predominantly been rooted in multidimensional conceptualizations of masculinity stemming from Latin American culture [28, 29]. Moreover, research with both Hispanic [28, 29] and non-Hispanic [30] samples indicate positive and protective aspects of masculinity are endorsed more among men than aspects that contribute to risky drinking.

Variations in 2002 to 2019 trends across race and ethnicity within sex were also evident. The increase in frequency of alcohol use was steeper for Black early adult females compared to White females. Although frequency of drinking does not necessarily map onto excessive consumption, this uptick in drinking among Black females should be considered in the context of research showing that Black women are more likely than White women to experience harms related to alcohol consumption due to structural influences (e.g., racial discrimination) [31]. On the other hand, declines for Black early adult males were significantly steeper than for Hispanic and White males. Researchers have posited a “margin for error” hypothesis, which suggests that some members of minoritized racial and ethnic groups may consume less alcohol because of an increased probability of experiencing certain negative alcohol-related consequences [32]. For instance, compared to White men, Black men are disproportionately incarcerated, serve longer sentences while incarcerated [33], and are more likely to be seriously or fatally injured by law enforcement [34, 35]. Increasing the centering of such disparities in public discourse may be contributing to a decrease in behaviors (e.g., participating in drinking contexts) among individuals who are particularly at risk of these consequences (e.g., early adult Black men).

In terms of AUD symptoms, only the rise among White middle adult males was different than the decline among Black and Hispanic middle adults. Past research with NSDUH data (using composite of years 2005/2006 and 2013/2014) shows alcohol use is increasing among adults aged 50 and older [7]. Researchers assert this increase may be driven by an increase in both supply (e.g., greater availability and marketing) and demand (e.g., more adults living longer healthier lives) [36]. Rising rates of AUD symptoms among White middle adults are particularly concerning alongside reports of steep increases in alcohol-related “deaths of despair,” that is, mortality related to alcohol poisoning and cirrhosis in this group [37, 38].

### Limitations

The current study has several limitations. First, the NSDUH data are cross-sectional and do not allow for analyzing change over time among individuals. Second, we cannot draw conclusions about explanatory mechanisms underlying the significant within- and between-group effects in this study. Third, it was not statistically feasible to examine trends among members of other ethnoracial groups. Similarly, NSDUH participants self-report sex dichotomized as female and male, which may not capture the sample’s gender diversity. Further, the measure of past-month alcohol use is limited to a measure of frequency (i.e., number of drinking days) with no commensurate measure of quantity as the latter was not available in the NSDUH data before 2015. Finally, we were not able to disaggregate individuals from 30 to 64 years due to sample sizes and pre-existing NSDUH categories for age. We also did not examine individuals over 64 years old given the relatively low rates of alcohol use within each of the subgroups, although patterns of alcohol use may be changing among older adults as well [7].

## Conclusion

Identifying changes in alcohol use and severity of AUD symptoms at the intersection of ethnoracial identity, sex, and age among US adults has important implications for addressing concerning alcohol-related national trends. At a clinical level, it is essential to consider intersectionality among patients to better inform their provision of clinical care. First, our finding that frequency of alcohol use is increasing only among Black females suggests that these trends should continue to be monitored, and health messaging around alcohol use tailored to this population may be warranted. That both female and male White middle adults showed increases in AUD symptoms is concerning, particularly given evidence that there is a decline in treatment utilization among people with AUD symptoms [12]. Many of the changes identified in the current study were small at the individual level but indicate major changes at the population level in light of the vast numbers of people affected by AUD symptoms [5] and suggests that harm reduction and reduced consumption efforts should target large but distinct sociodemographic groups. While the current study reveals significant differences in alcohol use and AUD symptoms based on the intersection of ethnoracial identity, sex, and age group, future research should contend with the social determinants underlying these differences.

## Data Availability

NSDUH data are publicly available through the Substance Abuse and Mental Health Services Administration at https://www.samhsa.gov/data/data-we-collect/nsduh/datafiles.
